# Cytokine release syndrome: grading, modeling, and new therapy

**DOI:** 10.1186/s13045-018-0653-x

**Published:** 2018-09-24

**Authors:** Delong Liu, Juanjuan Zhao

**Affiliations:** 1 Department of Oncology, The First Affiliated Hospital of Zhengzhou University, Zhengzhou, 450052 China; 2 The Affiliated Cancer Hospital of Zhengzhou University and Henan Cancer Hospital, 127 Dongming Road, Zhengzhou, 450008 China

## Abstract

Genetically modified T cells that express a chimeric antigen receptor (CAR) are opening a new frontier in cancer immunotherapy. CAR T cells currently are in clinical trials for many cancer types. Cytokine release syndrome (CRS) and neurotoxicities (CAR-related encephalopathy syndrome, CRES) are major adverse events limiting wide deployment of the CAR T cell treatment. Major efforts are ongoing to characterize the pathogenesis and etiology of CRS and CRES. Mouse models have been established to facilitate the study of pathogenesis of the major toxicities of CAR T cells. Myeloid cells including macrophages and monocytes, not the CAR T cells, were found to be the major cells mediating CRS and CRES by releasing IL-1 and IL-6 among other cytokines. Blocking IL-1 or depletion of monocytes abolished both CRS and CRES, whereas IL-6 blocker can ameliorate CRS but not CRES. Therefore, both IL-1 and IL-6 are major cytokines for CRS, though IL-1 is responsible for CRES. It was also demonstrated in the mouse models that blocking CRS does not interfere with the CAR T cell antitumor functions. We summarized new developments in the grading, modeling, and possible new therapeutic approaches for CRS and CRES in this review.

## Background

Genetically modified T cells that express a chimeric antigen receptor (CAR) are opening a new frontier in cancer immunotherapy [1–6]. Clinical trials of CAR T cells have been reported for many cancer types throughout the world [7–20]. Two types of CD19-directed CAR T cells, tisagenlecleucel and axicabtagene ciloleucel, have been approved for treatment of refractory/relapsed (R/R) non-Hodgkin lymphoma, whereas tisagenlecleucel was also approved for treatment of R/R acute lymphoid leukemia [15, 21–23].

Currently, the structure of a CAR contains an antigen-recognition domain, a transmembrane domain, a costimulatory segment, and a signaling domain [1, 24–26]. A single-chain fragment of variable region (scFv) replaces the conventional T cell antigen recognition domain. Therefore, the scFv confers the specificity of the engineered CAR T cells. More sophisticated CARs are being engineered, leading to more flexibility and controllability [27–29].

CAR T cells currently are in clinical trials for both hematological malignancies and solid tumors [11, 30–39]. Cytokine release syndrome (CRS) and neurotoxicities (CAR-related encephalopathy syndrome, CRES) are major adverse events limiting wide deployment of the CAR T cell treatment. Major efforts are ongoing to characterize the pathogenesis and etiology of CRS and CRES [40]. Mouse models of CRS have been established to facilitate the study of pathogenesis of the major toxicities of CAR T cells in animals. We summarized new developments in the grading, modeling, and therapeutic approaches for CRS and neurotoxicities in this review.

### CRS grading

CRS has characteristic clinical presentations, manifested typically with high fever, hypotension, hypoxia, and respiratory distress [41–44]. Organ dysfunctions such as liver transaminitis and renal insufficiency can occur. CRS toxicities occur as frequently as 90%, half of which is severe. Severe CRS complications are life-threatening and frequently require ICU care with vasopressors and/or ventilation support. The severities of CRS and neurotoxicities are directly related to the disease burden of ALL and the magnitude of CAR T cell expansion [22, 45, 46].

Several CRS grading systems have been proposed (Table 1) [41–44]. The CTCAE grading of CRS was initially developed for CRS related to blinatumomab infusion. The Lee and Neelapu grading systems allowed low-dose vasopressors and low requirement for oxygen (< 40%) as grade 2, whereas the Penn grading scale placed those to grade 3 when IV fluid resuscitation, any vasopressors, and/or oxygen is required. Clinically, it is more practical to place those who require any vasopressors to grade 3 (severe) since ICU care is required in most hospitals in such situation, whereas those who only require low-flow oxygen (mild hypoxia) and fluid resuscitation (mild hypotension) belong to grade 2. The different CRS grading system may have accounted for the reported differences in the rate of severe grades 3–4 CRS in different clinical trials [22, 47].

**Table 1:** CRS grading scales: Penn grading scale, CTCAE v4.0, 2014 Lee et al. scale, and MDACC grading

	Penn grading scale	CTCAE v4.0	2014 Lee et al.	MDACC
Grade 1	Mild reaction: treated with supportive care such as antipyretics and antiemetics	Mild reaction; infusion interruption not indicated; intervention not indicated	Symptoms are not life-threatening and require symptomatic treatment only, e.g., fever, nausea, fatigue, headache, myalgias, and malaise	Temperature ≥ 38 °C (fever) or grade 1 organ toxicity
Grade 2	Moderate reaction: some signs of organ dysfunction (e.g., grade 2 creatinine or grade 3 LFTs) related to CRS and not attributable to any other condition. Hospitalization for management of CRS-related symptoms, including fevers with associated neutropenia and need for IV therapies (not including fluid resuscitation for hypotension)	Therapy or infusion interruption indicated but responds promptly to symptomatic treatment (e.g., antihistamines, NSAIDs, narcotics, IV fluids); prophylactic medications indicated for ≤ 24 h	Symptoms require and respond to moderate intervention. Oxygen requirement < 40% or hypotension responsive to fluids or low-dose pressors or grade 2 organ toxicity	Systolic blood pressure < 90 mmHg (hypotension) but responds to IV fluids or low-dose vasopressors or needing oxygen(FiO2 < 40%) for SaO2 > 90% (hypoxia) or grade 2 organ toxicity
Grade 3	More severe reaction: hospitalization required for management of symptoms related to organ dysfunction, including grade 4 LFTs or grade 3 creatinine related to CRS and not attributable to any other conditions; this excludes management of fever or myalgias; includes hypotension treated with IV fluids (defined as multiple fluid boluses for blood pressure support) or low-dose vasopressors, coagulopathy requiring fresh frozen plasma or cryoprecipitate or fibrinogen concentrate, and hypoxia requiring supplemental oxygen (nasal cannula oxygen, high-flow oxygen, CPAP, or BiPAP). Patients admitted for management of suspected infection due to fevers and/or neutropenia may have grade 2 CRS	Prolonged reaction (e.g., not rapidly responsive to symptomatic medication and/or brief interruption of infusion); recurrence of symptoms following initial improvement; indicated for clinical sequelae (e.g., renal impairment, pulmonary infiltrates)	Symptoms require and respond to aggressive intervention. Oxygen requirement ≥ 40% or hypotension requiring high-dose or multiple pressors or grade 3 organ toxicity or grade 4 transaminitis	Systolic blood pressure < 90 mmHg (hypotension) and needs high-dose or multiple vasopressors or needing oxygen(FiO2 ≥ 40%) for SaO2 > 90% (hypoxia) or grade 3 organ toxicity or grade 4 transaminitis
Grade 4	Life-threatening complications such as hypotension requiring high-dose vasopressors and ^a^ hypoxia requiring mechanical ventilation	Life-threatening consequences; pressor or ventilator support indicated	Life-threatening symptoms. Requirements for ventilator support or grade 4 oxygen toxicity (excluding transaminitis)	Life-threatening hypotension or needing ventilator support or grade 4 oxygen toxicity (excluding transaminitis)

*BiPAP* bilevel positive airway pressure, *CPAP* continuous positive airway pressure therapy, *CRS* cytokine release syndrome, *CTCAE* Common Terminology Criteria for Adverse Events, *IV* intravenous, *LFT* liver function test, *NSAID* nonsteroidal anti-inflammatory drug, *FiO*_*2*_ fraction of inspired oxygen, *SaO*_*2*_ arterial oxygen saturation

^a^See specific definition of high-dose vasopressors [41]

Cytokine storm appears to be a distinct entity [41], even though clinical manifestations are similar to those of CRS. Cytokine storm appears to be mainly a result of non-specific T cell activation and occurs typically early in the course, shortly after CAR T cell infusion. The major cytokines in cytokine storm are TNF and IFN gamma [41]. Steroids are usually the first choice of therapy. CRS is usually the direct consequence of CAR T cell activation upon engagement of CAR with specific antigens. Therefore, CRS onset can vary in different diseases as well as with different CARs. For CTL019 clinical trials, the CRS onset timing ranged from 1 to 71 days [41], though most cases occurred between 1 and 14 days. The CRS onset timing in these CTL019 (tisagenlecleucel) trials varies according to the severities, with grades 1–3 CRS onset between a median of 8 and 9 days, whereas grade 4 CRS onset occurred at a median of 1 day (range 1–8 days) [8, 41, 48]. The median duration of CRS was reported to be between 5 and 12 days (range 1–23 days) [41]. CRS onset appears to be earlier in lymphoma trials. The key cytokine mediator is IL-6. Tocilizumab, the IL-6 receptor antibody, is the first-line of therapy.

Compartmental CRS (C-CRS) has been reported in a patient with advanced ovarian cancer treated with mesothelin-targeted CAR T cells [49]. In this case, elevation of IL-6 and accumulation of CAR T cells were seen in the pleural fluid. Tocilizumab therapy was effective. C-CRS was also reported in the brain, where IL-6, IFN gamma, and CAR T cells were significantly higher in the CSF than in the blood [50]. High-dose methylprednisolone successfully alleviated the cerebral CRS.

### CRS modeling

#### Establishment of CRS mouse models

CRS remains as a major life-threatening complication with complex pathogenesis. To further study and to better therapy of CRS, two mouse models have been reported [51, 52]. One model used SCID-beige mouse [51]. Raji tumor cells were injected intraperitoneally and allowed to grow 3 weeks. The tumor cells formed vascularized solid masses. Thirty million human 1928z CAR T cells were used to target the B leukemia cells and solicit CRS, which occurred 2–3 days after CAR T infusion. The mouse model recapitulated the constellation of CRS symptoms and signs. Serum cytokines of human origin, IL-2, IL-3, IL-6, IFN gamma, and GMCSF were elevated in CRS.

The other mouse model used humanized NSG mice which produce human cytokines of IL-3, stem cell factor, and GM-CSF to support and enhance hematopoiesis from human stem cells [52]. Patient-derived ALL cell line, ALL-CM, was used as targets for CD19.BBz and CD19.28z CAR T cells. After the CAR T cell challenge, a violent systemic inflammatory syndrome occurred, recapitulating severe human CRS.

#### Macrophages and monocytes are mediators of CRS

Using the SCID-beige mouse model, Sadelain’s group chased after the source of IL-6 released during CRS. The source was traced to macrophages. It was discovered through RNA-seq analysis that the main source of IL-6 came from the macrophages and monocytes. Production of major inflammatory cytokines was confined to the site of CAR T cell-tumor tissue colocalization in the peritoneum.

In the humanized NSG model, monocytes were found to be the main source of CRS cytokines. Monocyte depletion essentially abolished CRS and protected mice from CRS-induced lethality.

These two separate mouse model systems independently confirmed that macrophages and monocytes are the direct mediators of CRS, not CAR T cells.

#### IL-1 is the primary cytokine for CRS and CRES

It is well known that IL-6 is elevated in patients with CRS, and IL-6 receptor antagonist, tocilizumab, is an effective therapy for CRS. This was reproducible in the mouse models. However, it was demonstrated in both models that IL-1 is the primary cytokine responsible for both CRS and CRES. Blocking IL-1 receptor with anakinra effectively abolished CRS as well as neurotoxicities, whereas blockage of IL-6 with tocilizumab abrogated only CRS, not neurotoxicities. It is known that macrophages/monocytes produce IL-1 upon activation, and IL-1 in turn can elicit production of IL-6 by the macrophages/monocytes. Sadelain’s group further demonstrated that by engineering CAR T cells to constitutively express an IL-1 receptor antagonist, CRS was prevented [51].

#### Hypotension associated with CRS is induced by nitrogen oxide (NO)

It was further demonstrated in the SCID-beige mouse model that NO synthetase (NOS) was induced by IL-1 and IL-6 during CRS. As a result, NO production by macrophages was increased, leading to hypotension which is a major life-threatening complication of CAR T-induced severe CRS. Using NOS inhibitors, L-NIL or 1400W, the investigators were able to alleviate the systemic toxicities and reduce mortality secondary to severe CRS. These findings provided new evidence for CRS pathophysiology and a new means to possibly prevent and effectively treat severe CRS. Inhibition of NOS activity may significantly reduce CRS severity and possibly eliminate or reduce requirements for ICU care of CRS toxicity.

NOS was downregulated by blocking either IL-1 or IL-6, leading to reduced mortality in the mouse model. This confirms that upregulation of NOS is a unifying mechanism for both IL-1- and IL-6-induced severe CRS. However, simultaneous blockade of IL-1 and IL-6 does not further reduce the fraction of NOS+ macrophages [51]. This implies that clinically only blocking one of the two cytokines is needed to abrogate CRS.

#### Blocking CRS does not reduce CAR T functions

There have been concerns that treatment for CRS could reduce the activity of CAR T cells. Therefore prophylaxis for CRS by steroids and IL-6 blockage has not been encouraged currently in the clinical practice.

This question was specifically addressed in the mouse models. Using the IL-1R antagonist producing CAR T cells, it was demonstrated that blocking CRS did not reduce the 1928z CAR T cell antitumor efficacy. Therefore, preventing CRS does not affect CAR T cell functions. The preservation of antitumor efficacy was also demonstrated where similar leukemia clearance by CAR T cells in the humanized HuSGM3 mice treated with either tocilizumab or anakinra [52] occurred. These observations suggest that clinically it may be preferred to apply tocilizumab as early as prophylaxis for CRS so that morbidity and mortality from CART-related toxicities (CARTox) can be significantly reduced.

#### IL-1 mediates both CRS and CRES

In the HuSGM3 mouse model, occurrence of lethal neurological syndrome with paralysis and seizures was unexpectedly observed in the mice that received either vehicle or tocilizumab. Meningeal thickening together with human macrophage infiltration in these mice was demonstrated. It was further shown that the meningeal thickening was prevented by administration of anakinra, but not by tocilizumab, even though CRS was effectively abolished by both agents. It was concluded that only anakinra prophylaxis prevented both CRS and neurotoxicity and increased mice survival. This study established that both IL-1 and IL-6 are causal cytokines for CRS, though IL-1 is responsible for severe neurotoxicities (CRES).

### New approach for clinical management of CRS and CRES

Currently, for early-onset CRES, such as confusion, hallucination, aphasia, and seizure, steroids are generally used first. For CRS characterized by high fever, hypotension, and hypoxia, tocilizumab is infused over an hour. The dosage is calculated according to patients’ weight. For patients’ weight less than 30 kg, the dosage is 12 mg/kg, whereas the dosage is 8 mg/kg when the weight is more than 30 kg (for official recommendations, please refer to the full prescribing information at https://www.gene.com/download/pdf/actemra_prescribing.pdf). The treatment can be repeated when clinically necessary, up to three more doses, at least 8 hours apart.

With the new discovery of IL-1 being a major cytokine for both CRS and CRES from the mouse models, it is reasonable to consider anakinra, an IL-1R antagonist, for CAR T-induced toxicities (CARTox), namely, CRS and CRES. Since blocking CARTox has no adverse effect on CAR T antitumor efficacy, it is reasonable to use anakinra or tocilizumab for CRS prophylaxis. Anakinra may be the preferred agent for prophylaxis since it is effective to prevent both CRS and CRES. Tocilizumab may be used to prevent CRS but not effective for prophylaxis of neurotoxicities. In the SCID-beige mouse model, NOS inhibitors were shown to reduce systemic toxicities of CRS. Therefore, these agents may have potential for the management of CARTox and reduce the use of vasopressors. However, these were reports from animal models. Clinical trials are needed to confirm the effect of anakinra for both CRS and CRES as well as NOS inhibitors. Once it is confirmed clinically, a significant reduction in CARTox is expected in the near future. Clinical confirmation of these significant findings from mouse models is urgently needed.

Currently, tocilizumab is being studied in a clinical trial for prophylaxis of CRS (Table 2). Since it has been observed that CRS severity is directly related to leukemia burden in ALL and severe CRS occurs earlier, timing of tocilizumab therapy is being studied in a two-cohort open-label pilot study (Table 2). Tocilizumab is scheduled to start earlier in high-leukemia-burden patients.

**Table 2:** Clinical trials of tocilizumab for cytokine release syndrome

NCT no.	Trials	Conditions	Interventions	Locations
03533101	Tocilizumab for Cytokine Release Syndrome Prophylaxis in Haploidentical Transplantation	•Cytokine release syndrome •Stem cell transplant complications	•Drug: tocilizumab	•Hospital Universitario Dr. Jose E Gonzalez UANL, Monterrey, Nuevo Leon, Mexico
03275493	Humanized CD19 CAR-T Cells With CRS Suppression Technology for r/r CD19+ Acute Lymphoblastic Leukemia	•Acute lymphoblastic leukemia •CD19 positive •Relapse •Refractory	•Biological: humanized CD19 CAR-T cells •Biological: humanized CD19 CAR-T cells with CRS suppression technology	•The First Affiliated Hospital of Soochow University, Suzhou, Jiangsu, China
02906371	Study of the Tocilizumab Optimization Timing for CART19 Associated Cytokine Release Syndrome	•Lymphoblastic leukemia, acute, childhood	•Drug: tocilizumab •Biological: CART 19	•Children’s Hospital of Philadelphia, Philadelphia, PA, USA

### Conclusions

Currently, there are several CRS scoring models (Table 1). These were used for different clinical trials. It is ideal to have a consensus and to unify the scoring systems. There is no CRES-specific scoring system available yet. In addition to prophylaxis, new CAR design is needed to better control and adjust the CAR T cell engagement of target cancer cells. SUPRA CAR design offers a flexible, switchable, and more adjustable construct as demonstrated in the mouse model [53]. This SUPRA CAR design may bring more control in CRS. Clinical trial of the SUPRA CAR T cells is clearly needed.

Blinatumomab is a bispecific T cell engager (BiTE) approved for therapy of acute lymphoblastic leukemia [54–56]. Bispecific antibodies are associated with the similar profile of CRES and CRS seen in CAR T therapy, albeit at a lower incidence and with less severity [57–59]. Since the pathophysiology of the CRS and CRES incited by blinatumomab is similar to CARTox, and IL-6 blockade is equally effective for CRS, it is possible that macrophage-produced IL-1 plays a major role in the toxicities [42]. Therefore, anakinra may be a therapeutic and prophylactic option for the CRS and CRES associated with blinatumomab. Clinical trial of anakinra is indicated.

A variety of systemic inflammatory syndromes, such as acute respiratory distress syndrome (ARDS) and macrophage activation syndrome/hemophagocytic lymphohistiocytosis (HLH), have similar clinical manifestations with high fever, hypoxia, and hypotension. The findings from the CRS models appear to be provocative for implications of management for ARDS and HLH. Etanercept, a soluble TNFa receptor, has been used successfully for CRS therapy [42]. It is possible that IL-1 and/or IL-6 is also a major cytokine for the syndromes [60–62]. Further studies on use of anakinra and/or tocilizumab in these syndromes would be indicated. A clinical trial of tocilizumab for HLH is underway (NCT02007239).

## Abbreviations

CAR: Chimeric antigen receptor
CRES: CAR-related encephalopathy syndrome
CRS: Cytokine release syndrome
